# Supplementary material for: Empirical methods for controlling false positives and estimating confidence in ChIP-Seq peaks
Source: BMC Bioinformatics. 2008 Dec 5;9:523. doi: 10.1186/1471-2105-9-523 (PMC2628906; doi:10.1186/1471-2105-9-523)
Supplement: Additional File 1 — A variety of html documents from the USeq web site detailing the available applications, their best usage, output file type descriptions, command line menus, etc. [file 1471-2105-9-523-S1.zip › USeqUserGuides/JavaDoc.zip!/JavaDoc/util/bio/wrappers/class-use/BlastResult.html]

Uses of Class util.bio.wrappers.BlastResult


|  |  |  |
| --- | --- | --- |
| **Overview** \| **Package** \| **Class** \| **Use** \| **Tree** \| **Deprecated** \| **Index** \| **Help** | |  |
| PREV   NEXT | **FRAMES**    **NO FRAMES**     **All Classes** |  |


---


## **Uses of Class util.bio.wrappers.BlastResult**

| Packages that use BlastResult | |
| --- | --- |
| **bioroot** |  |
| **util.bio.wrappers** | Wrappers for other bio applications. |

Uses of BlastResult in bioroot

| Methods in bioroot with parameters of type BlastResult | |
| --- | --- |
| `static java.lang.String` | `Search.blastResultToHTML(BlastResult res)` |
| `static java.lang.StringBuffer` | `Search.makeBlastSummaryTable(BlastResult[] res)`             Returns a summary HTML table, hotlinked id, name, score, expect |

Uses of BlastResult in util.bio.wrappers

| Methods in util.bio.wrappers that return BlastResult | |
| --- | --- |
| `static BlastResult[]` | `SimpleBlast.parseBlastGeneral(java.lang.String[] data)`             Parses blast output from blastall for dna into BlastResult objects. |

---


|  |  |  |
| --- | --- | --- |
| **Overview** \| **Package** \| **Class** \| **Use** \| **Tree** \| **Deprecated** \| **Index** \| **Help** | |  |
| PREV   NEXT | **FRAMES**    **NO FRAMES**     **All Classes** |  |


---
